# The Role of Electrical Impedance Tomography for Management of High-Risk Pulmonary Embolism in a Postoperative Patient

**DOI:** 10.3389/fmed.2021.773471

**Published:** 2021-11-19

**Authors:** Xinchen Wang, Hua Zhao, Na Cui

**Affiliations:** Department of Critical Care Medicine, Peking Union Medical College Hospital, Chinese Academy of Medical Science and Peking Union Medical College, Beijing, China

**Keywords:** electrical impedance tomography, pulmonary embolism (MeSH), postoperative, beside, anticoagulation, bleeding

## Abstract

Electrical impedance tomography (EIT) is a non-invasive, radiation-free and bedside imaging tool that is widely used for real-time monitoring of lung ventilation. Recently, it has been proposed for use in quantitative assessment of regional lung perfusion with hypertonic saline bolus injection and consequently for pulmonary embolism (PE) detection. Here, we present a case of high-risk PE in a postoperative patient, in which EIT monitoring provided us with useful information for diagnosis and decision-making, especially with the challenge of anticoagulation and risk of bleeding.

## Introduction

Pulmonary embolism (PE) is the third most frequent acute cardiovascular syndrome worldwide after myocardial infarction and stroke (1). High-risk PE, as indicated by the presence of hemodynamic instability, is a life-threatening situation that requires an immediate emergency diagnostic and therapeutic strategy at its occurrence (2). Furthermore, rapid and bedside methods to monitor pulmonary perfusion are actual demands for clinical decision-making during the management of high-risk PE. For example, postoperative patients with a complication of high-risk PE are more likely to experience hemorrhage during thrombolysis or large-dose anticoagulant therapy, necessitating the discontinuation of the anticoagulant therapy. However, for such patients, discontinuing intense anticoagulant therapy could lead to progression of the embolism and cause death. In this circumstance, rapid and bedside methods to monitor pulmonary perfusion will be especially helpful to gather more convincing and direct indications to make decisions.

Electrical impedance tomography (EIT) is a non-invasive, radiation-free imaging tool that is widely used for real-time monitoring of lung ventilation. EIT image reconstruction is based on the estimation of the resistivity changes that occur across the lungs with breathing (3). In several recent clinical studies, it has been proposed for use in quantitative assessment of regional lung perfusion with hypertonic saline bolus injection and consequently for PE detection (4, 5). Here, we present a case of EIT-guided management of high-risk PE in the intensive care unit (ICU) of Peking Union Medical College Hospital.

## Case Description

**Figure 1:**
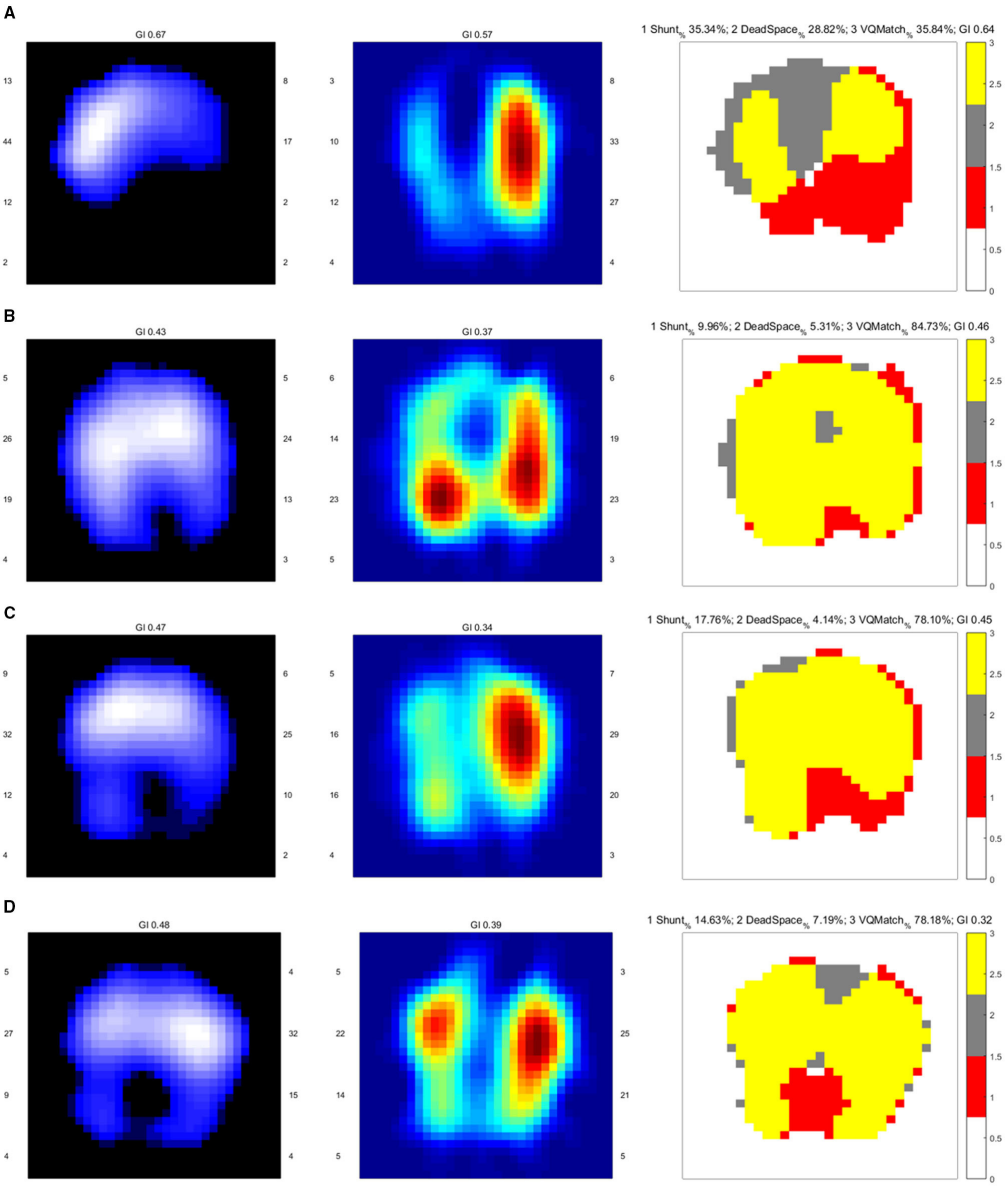
EIT images of the functional ventilation distribution (dark blue areas indicated low ventilated regions and white areas indicated high ventilated regions), functional perfusion distribution (red areas indicated high-perfusion regions and blue areas indicated low-perfusion regions), and distribution of the regional ventilation/perfusion ratios (Ventilated regions were defined as pixels with impedance changes higher than 20% of the maximum tidal impedance variation in the functional ventilation image. Perfused regions were defined as pixels higher than 20% of the maximum bolus-related impedance change in the functional perfusion image. Gray areas indicated regions with high ventilation and low perfusion. Red areas indicated low ventilation and high perfusion regions. Yellow areas indicated good ventilation-perfusion matching). **(A)** On admission, there was a significant ventilation defect in the dorsal lung and perfusion defect in the right lung, leading us to perform CTPA for PE confirmation. The ventilation defect was later treated with lung recruitment and tracheal suction. **(B)** Ventilation improved after lung recruitment and tracheal suction. Regional perfusion in the right lung was restored after thrombolysis. **(C)** On Day 5, there was a ventilation defect in the left dorsal lung, with a significant shunt observed in the image of the ventilation/perfusion (V/Q) ratio distribution. **(D)** On Day 12, there were ventilation defects in the dorsal lung. The lung perfusion demonstrates symmetric in both lungs.

**Figure 2:**
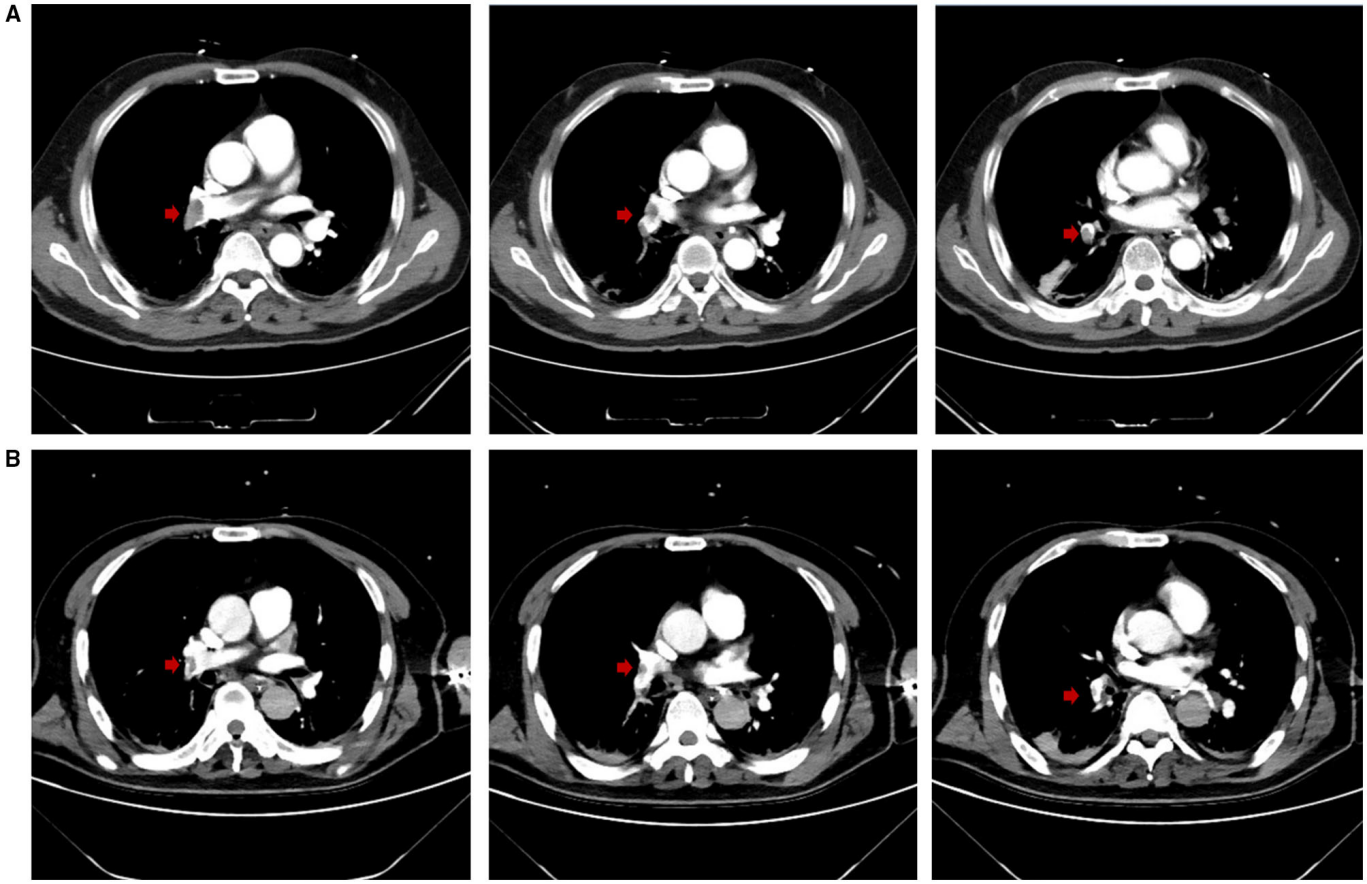
**(A)** CTPA at admission demonstrated multiple embolisms in the right pulmonary trunk and right pulmonary artery branches (embolisms in the right pulmonary artery branches not shown). Red arrows highlight the locations of the emboli. **(B)** On Day 12, CTPA revealed reduced embolisms in the right pulmonary trunk and right pulmonary artery branches, and improved artery visualization could be observed. Red arrows highlight the locations of the emboli.

A 64-year-old man with a history of bladder cancer and prostate cancer, who had successfully undergone laparoscopic radical cystectomy and ileum conduit urinary diversion during this admission, was referred to the ICU due to sudden hypoxemia and extreme dyspnea on the sixth postoperative day. The patient had a respiratory rate (RR) of 40 times per minute and pulse oxyhemoglobin saturation (SpO_2_) of 87% under 10 L/min oxygen supplied by an oxygen storage mask. In addition, the patient had a heart rate (HR) of 114 b.p.m., blood pressure (BP) of 149/73 mmHg, and body temperature of 37.6°C. After sedation, intubation was performed on the patient, and mechanical ventilation was then administered (VC mode, VT 400 ml, PEEP 5 cmH2O, FiO_2_ 40%). Continuous infusion of norepinephrine (NE) at a rate of around 1 ug/kg/min was given to the patient to maintain an MAP of 80 mmHg. Continuous infusion of Cisatracurium, a muscle relaxant, at 3 mg/h was later given to control the excessive inspiratory effort. Laboratory evaluation revealed an elevated D-D dimer concentration of 28.5 mg/L (Other lab results shown in Table 1). The Wells score was 7 (HR ≥ 100 b.p.m., surgery, active cancer, alternative diagnosis less likely than PE) and PE was strongly suspected. After informed consent was obtained, bedside EIT with hypertonic saline (10%) bolus infusion was performed to assess the regional pulmonary perfusion for PE detection. From the EIT pulmonary perfusion images, there was a significant perfusion defect in the right lung with dead space accounting for 28.82% (Figure 1A). Considering the life-threatening emergency of the suspected PE that requires urgent thrombolysis after its confirmation, we took the risk to transfer the patient for a CT pulmonary angiography (CTPA) examination. The CTPA showed multiple embolisms in the right pulmonary trunk, right pulmonary artery branches, and left artery branches (Figure 2A). During the screening of deep venous thrombosis, multiple calf muscle venous thrombosis was observed in the lower right extremities, which could be the source of PE.

**Table 1:** Data of PE-related monitoring at different stages of PE recovery.

	**Day 1**	**Day 2**	**Day 5**	**Day 12**
Milestone	Admission	After thrombolysis	Oxygenation improved	Relief from dyspnea and weaning
Heart rate, b.p.m.	114	76	104	96
MAP, mmHg	67	79	80	86
NE, ug/kg/min	1	0.2	0.413	0
CVP, mmHg	10	7	5	5
Pv-aCO2, mmHg	5.4	0.5	5.6	6.4
ScvO_2_, %	69.4	84.2	65.4	72.6
Lac, mmol/L	2	0.7	0.8	0.5
WBC, 10^9/*L*^	7.70	7.98	11.52	13.77
P/F ratio, mmHg	156	161	291	347
D-Dimer, mg/L	28.5	318.5	4.92	11.37
Hb, g/L	129	122	93	88
Cr, mmol/L	152	161	133	272
cTnI, ug/L	0.051	0.065	0.027	<0.017
Respiratory conditions	PC 13 cmH2O, PEEP 6 cmH2O, FiO_2_ 45%, f 20 c.p.m.	PC 12cmH2O, PEEP 6 cmH2O, F FiO_2_ 45%, f 18 c.p.m.	PS 12cmH2O, PEEP 10 cmH2O, FiO_2_ 40%, RR 13 c.p.m.	Venturi, FiO_2_ 31%, RR 15 c.p.m.
MV	11.0 L/min	10.1 L/min	7.6 L/min	-
**Percentage of pulmonary perfusion by EIT (right vs. left), %**				
ROI 1	3 vs. 8	6 vs. 6	5 vs. 7	5 vs. 3
ROI 2	10 vs. 33	14 vs. 19	16 vs. 29	22 vs. 25
ROI 3	12 vs. 27	23 vs. 23	16 vs. 20	14 vs. 21
ROI 4	4 vs. 4	5 vs. 3	4 vs. 3	5 vs. 5
Shunt, %	35.34	9.96	17.76	14.63
Dead space, %	28.82	5.31	4.14	7.19
V/Q match, %	35.84	84.73	78.10	78.18
LU findings	L6: tissue-like signs	L6: tissue-like signs	L6 and R6: tissue-like signs	R4, L6, and R6: tissue-like signs
CT scan findings	Consolidation in the lower left lung	-	-	Consolidation in the lower bilateral lung.
TVR, m/s	3.4	2.9	2.0	Poor ultrasonic conditions.
CO, L/min	8.1	4.8	5.3	Poor ultrasonic conditions.
Events	Thrombolysis	Continuous anticoagulant therapy to achieve the APTT at 50–70 s.	Severe bleeding of the lower gastrointestinal tract. Suspension of anticoagulant therapy.	Suspension of anticoagulant therapy.

**Figure 3:**
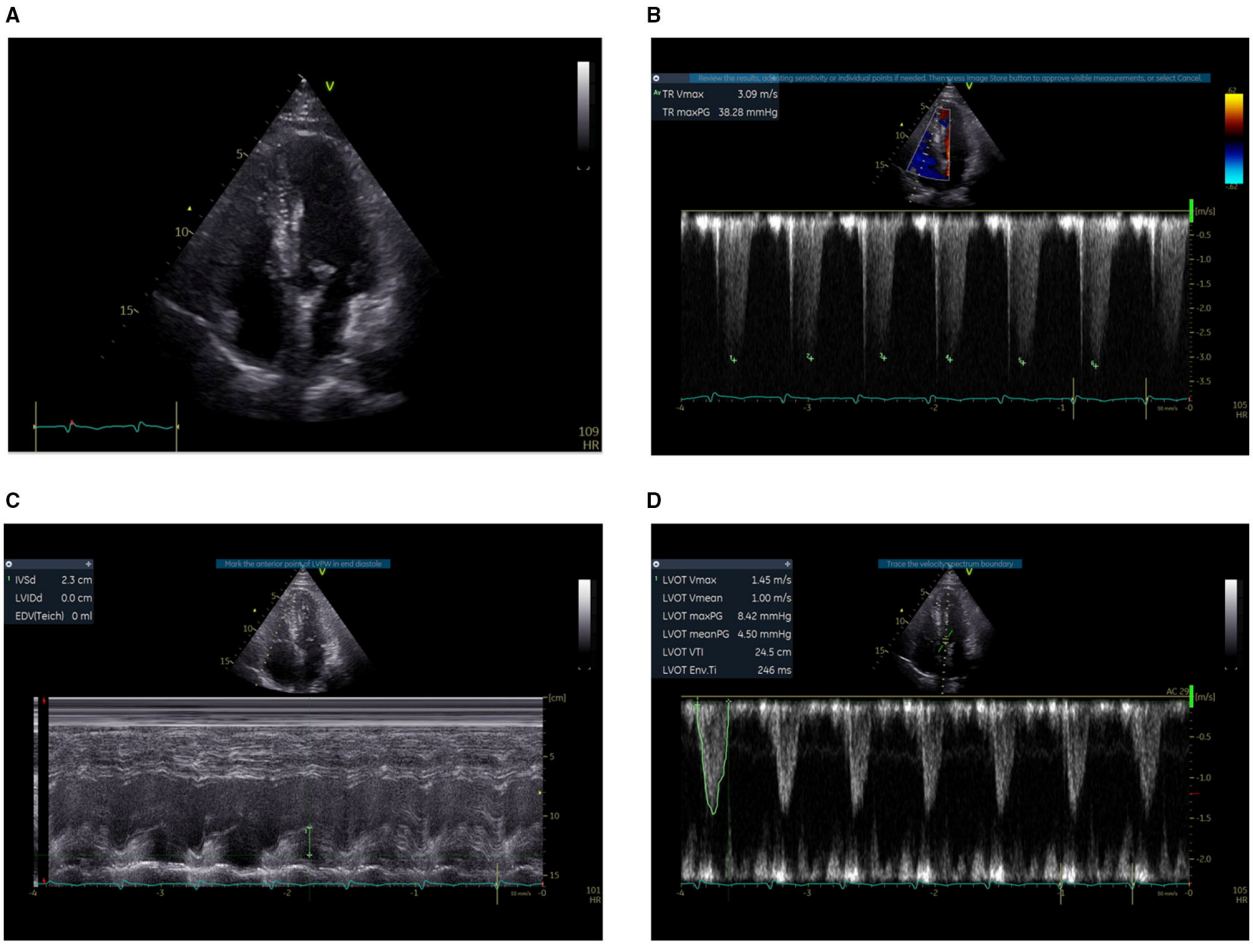
Bedside TTE before thrombolysis. **(A)** The right ventricle was not clearly displayed, with an estimated ratio of less than 1:1 between the RV and the LV. **(B)** The TVR was 3.09 m/s. **(C)** The systolic function of the RV measured by TAPSE was 2.3 cm. **(D)** The LVOT VTI was 24.5 at an HR of 105 b.p.m. and the estimated CO was 8.07 L/min.

Since the diagnosis of PE, the PESI score of the patient was 194 (64 years old, male sex, cancer, pulse rate ≥ 110 b.p.m., systolic BP < 100 mmHg, RR > 30 c.p.m., arterial oxyhemoglobin saturation < 90%) with risk strata of Class V (very high mortality risk) (6). Bedside transthoracic echocardiography (TTE) was performed to evaluate the right ventricle function in particular. The systolic D-shaped left ventricle was observed from the parasternal short-axis view. The right ventricle (RV) was not clearly displayed in the apical four-chamber section, with an estimated ratio of less than 1:1 between the RV and the left ventricle (LV). The tricuspid valve regurgitation (TVR) was 3.09 m/s. The systolic function of RV measured by TAPSE was 2.3 cm. The left ventricular outflow tract (LVOT) velocity-time integral (VTI) was 24.5 at an HR of 105 b.p.m., and the estimated cardiac output (CO) was 8.07 L/min (Figure 3). Thrombolysis (alteplase 50 mg) was initiated immediately, three hours after which continuously-infused heparin was given to the patient to achieve the activated partial thromboplastin time (APTT) at 50–70 s.

**Figure 4:**
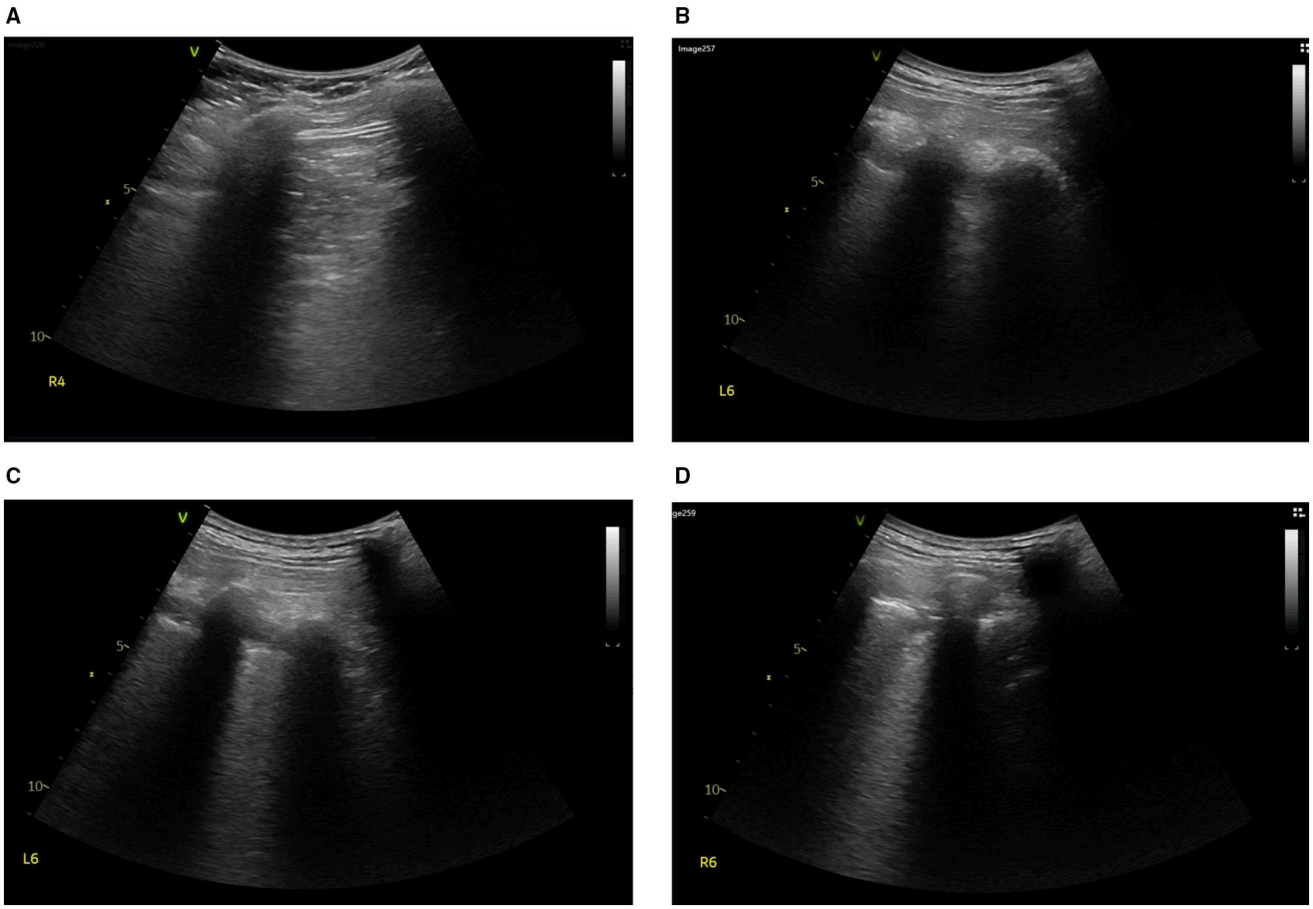
On Day 5, lung ultrasound was performed to find the cause of ventilation defect in the left dorsal lung. Tissue-like signs were found in the region of **(A)** R4, **(B,C)** L6, and **(D)** R6.

After thrombolysis, another EIT with hypertonic saline bolus infusion was performed to observe lung perfusion. The EIT image showed that regional perfusion was restored in the right lung (Figure 1B). Meanwhile, bedside TTE revealed that the TVR was reduced to 2.9 m/s, and the RV did not further enlarge. All the evidence supported the efficacy of thrombolysis and anticoagulant therapy, thus the anticoagulant therapy was continued, with the patient's condition closely monitored. On the fifth day after thrombolysis (and since ICU admission), the patient experienced massive lower gastrointestinal bleeding, with his hemoglobin levels decreasing from 108 g/L to 88 g/L. The patient was in shock due to an estimated blood loss of 800 ml in one day, with an HR of 104 b.p.m. and MAP of around 80 mmHg, receiving a continuous infusion of NE at 0.413 ug/kg/min. According to EIT, the pulmonary perfusion had recovered and appeared bilaterally symmetric on the same day, while the hypoxemia of the patient improved from a P/F ratio of 161 to 291 mmHg. Therefore, the coagulant therapy was stopped. Meanwhile, the pulmonary ventilation image of EIT revealed a new ventilation defect in the left dorsal lung, with a significant shunt accounting for 17.76% in that area (Figure 1C). Combined with the lung ultrasound findings, tissue-like signs were detected in the L6 and R6 regions using a six-zone scanning protocol (Figure 4), while lung sliding and A-lines were observed in the other regions.

It was concluded that there could be consolidation in the dorsal region of bilateral lungs. Therefore, intermittent prone positioning and physical vibration were performed on the chest to help expel the sputum, and re-expand the collapsed lung. The bleeding did not stop until Day 10. On Day 12, the anticoagulant therapy was still suspended due to the high risk of bleeding. However, the patient was relieved from dyspnea, the oxygenation index improved further to a P/F ratio of 347 mmHg, and weaning had been successfully processed. The patient was taken for another CTPA examination, after which we performed EIT with hypertonic saline bolus infusion. CTPA revealed that the emboli in the right trunk, right pulmonary artery branches, and left artery branches had decreased in size, and the pulmonary artery was more visualized in the angiography (Figure 2B). The pulmonary perfusion image of EIT revealed homogeneous perfusion in both the left and right lungs, indicating that the pulmonary perfusion of the lung did not worsen without anticoagulation (Figure 1D). Based on this information, we decided to continue closely monitoring the patient's condition without anticoagulation. The following day, the patient was extubated. The monitor revealed that the RR was 17 c.p.m., SpO2 was 99% under 2 L/min oxygen supplied by a nasal cannula, HR was 80-90 b.p.m., and BP was 148/76 mmHg with no vasopressor. The patient was relieved from hypoxemia and dyspnea, and recovered from hemorrhage. On Day 15, he was finally transferred to the general ward. The anticoagulation therapy was not restarted until Day 20, and the respiratory condition of the patient remained stable.

## Discussion

The 30 day mortality of high-risk PE (with PESI Class V or sPESI ≥ 1 point) is between 10.9 and 24.5% (2). In this case, EIT was used as a valuable bedside tool for clinical decision-making during the management of high-risk PE. As a result, the patient was successfully cured of the vital respiratory failure and circulatory shock caused by PE. Although CTPA has long been the traditional confirmation test of PE (3), for critical PE patients who are frequently in shock and have severe hypoxemia, the potential danger of transferring patients for CTPA has put clinicians and patients in a dilemma. A higher risk of contrast-induced nephropathy caused by CTPA is also a concern for patients with acute or chronic kidney disease complications. The EIT method for generating pulmonary perfusion images is a first-pass kinetic approach that involves administering a bolus of 10 ml hypertonic saline as a contrast agent through a central venous catheter during a breath-hold (7). It is inexpensive, portable, radiation-free, with minimal risk, and not restricted by the patients' posture. As a substitute for CTPA, its results have been proven to be highly reproducible (8), and its diagnostic efficacy of PE has recently been reported. A recent prospective observational study between PE and non-PE patients with acute respiratory failure has found that a higher dead space percentage, lower intrapulmonary shunt percentage, and lower V/Q match can predict the presence of PE. The cutoff value of 30.37% for dead space percentage resulted in a sensitivity of 90.9% and a specificity of 98.6% for the PE diagnosis (4).

In this case, when PE was highly suspected, bedside EIT was performed to assess regional pulmonary perfusion instead of transferring the patient who had severe dyspnea and hemodynamic instability for CTPA. The pulmonary perfusion image of EIT revealed a significant perfusion defect of the right lung and urged us to perform CTPA. This is the first case report that PE had been early diagnosed by EIT, which further triggered a CTPA confirmation test. The perfusion defect detected by EIT was consistent with the CTPA findings, although the percentage of dead space detected in this case was lower than the previous study (4). One possible explanation is that poor ventilation of the dorsal lung caused by pulmonary atelectasis resulted in a lower calculated dead space percentage. Thus, the diagnosis of PE cannot be excluded even with a lower dead space percentage in such patients. The ventilation defect detected in the right lung at the occurrence of PE was suspected to be regional atelectasis, due to severe respiratory stress of the patient. After restraining excessive respiratory effort with the muscle relaxant, tracheal suction and lung recruitment, the ventilation defect was quickly reduced, as evidenced by the second EIT examination (Figures 1A,B).

In this case, the first question we faced was whether thrombolysis was having a sufficient effect, especially since clinical manifestations did not change prominently after they occurred. The pulmonary perfusion image of EIT revealed key information on the improvement of pulmonary perfusion. Combined with the improved TTE results, it answered our question and determined continuous anticoagulant therapy. When the patient experienced life-threatening lower gastrointestinal bleeding on Day 5, the anticoagulant therapy had to be stopped. However, the patient was still at high risk of PE with a Wells score of 7, and having another attack of PE could be deadly. The following anticoagulant therapy after thrombolysis in PE treatment aims at reducing the embolic burden and relieving the vascular obstruction, which could be indicated by the stable bilaterally symmetry pulmonary perfusion by EIT. In this case, the stability of pulmonary perfusion acquired by EIT was found to be consistent with the continued improvement of the patient's oxygenation index, dyspnea, and weaning, suggesting bedside monitoring of pulmonary perfusion could be of much value. Although the value of EIT for PE diagnosis has been widely reported, this is the first time EIT has been used as a valuable tool for clinical decision-making on the adjustment of anticoagulant therapy in a postoperative high-risk PE patient with a high risk of bleeding. Furthermore, it should be noted that the assessment of pulmonary perfusion by EIT cannot be replaced by CTPA in terms of the functional evaluation of lung perfusion. Smaller emboli were still present in situ according to the follow-up CTPA, but there was no indication of changing pulmonary perfusion.

On Day 5, a prominent ventilation defect with a pulmonary shunt was discovered during EIT monitoring. Combined with the lung ultrasound findings, regional consolidation was highly suspected, which was later verified by the follow-up CT scan (Figure not shown). Following that, the targeted treatment was initiated to re-expand the collapsed lung. Despite pulmonary perfusion, assessment of pulmonary ventilation by EIT also plays a key role in the diagnosis and treatment of different causes of dyspnea and hypoxemia. Therefore, we diagnosed lung consolidation and initiated targeted treatment for lung consolidation. Although lung consolidation did not appear to improve based on the later EIT image on Day 12, due to EIT, the therapy was expanded to include a broader range of respiratory treatments, instead of being limited to only PE treatment.

The role of EIT monitoring in the management of postoperative high-risk PE is a classic case of its practicability in critical settings. When patients are at high risk of transfer for CTPA, EIT with hypertonic saline bolus infusion could aid in the diagnosis of PE. Furthermore, EIT could be an important indication of pulmonary perfusion to guide or back up the adjustment of anticoagulant therapy when faced with a conflict between anticoagulation and the risk of bleeding. In addition, when combined with perfusion, EIT evaluation of pulmonary ventilation can aid in the diagnosis of other respiratory conditions, for example, lung consolidation. However, we cannot ignore the disadvantages of the technique, including low-resolution, complex image reconstruction procedure, limited field of view, and incompatibility with certain patients, such as those with a pacemaker (7). In this case, breath-holding was found to be particularly difficult for patients with dyspnea. Furthermore, EIT is unable to reflect the exact anatomical location of PE and detect small emboli. Therefore, it could be misleading to use it as the only indication to decide against anticoagulant therapy. In this case, the therapeutical decision was made based on a variety of clinical data, including but not limited to EIT. A full understanding of EIT in as many aspects as possible is required for its application in the clinical scenarios.

In conclusion, EIT could provide useful information for clinical decision-making in the context of both pulmonary perfusion and pulmonary ventilation during the diagnosis and treatment of high-risk PE, especially with the challenge of anticoagulation and risk of bleeding, which may benefit critically ill patients in the future.

## Data Availability Statement

The raw data supporting the conclusions of this article will be made available by the authors, without undue reservation.

## Ethics Statement

Written informed consent was obtained from the individual(s) for the publication of any potentially identifiable images or data included in this article.

## Author Contributions

XW and HZ collected study data and drafted the present manuscript. NC revised the manuscript. All authors read and approved the final version of the manuscript.

## Funding

This work was supported by National Natural Science Foundation of China (No. 82072226), Beijing Municipal Science and Technology Commission (No. Z201100005520049), Non-profit Central Research Institute Fund of Chinese Academy of Medical Sciences (No. 2019XK320040), Tibet Natural Science Foundation (No. XZ2019ZR-ZY12(Z)), and Excellence Program of Key Clinical Specialty of Beijing in 2020 (No. ZK128001).

## Conflict of Interest

The authors declare that the research was conducted in the absence of any commercial or financial relationships that could be construed as a potential conflict of interest.

## Publisher's Note

All claims expressed in this article are solely those of the authors and do not necessarily represent those of their affiliated organizations, or those of the publisher, the editors and the reviewers. Any product that may be evaluated in this article, or claim that may be made by its manufacturer, is not guaranteed or endorsed by the publisher.

## Abbreviations

SpO2: pulse oxyhemoglobin saturation
b.p.m.: beats per minute
c.p.m.: counts per minute
VC: volume control
VT: tidal volume
PEEP: positive end-expiratory pressure
MAP: mean artery pressure
P/F: artery partial pressure of oxygen
FiO_2_: fraction of inspired oxygen ratio
ROI: region of interest
LU: Lung ultrasound
ScvO2: Central venous oxygen saturation.
